# Longitudinal assessment of HLA and MIC-A antibodies in uneventful pregnancies and pregnancies complicated by preeclampsia or gestational diabetes

**DOI:** 10.1038/s41598-017-13275-6

**Published:** 2017-10-19

**Authors:** Lorenz Küssel, Harald Herkner, Markus Wahrmann, Farsad Eskandary, Konstantin Doberer, Julia Binder, Petra Pateisky, Harald Zeisler, Georg A. Böhmig, Gregor Bond

**Affiliations:** 1 Department for Obstetrics and Gynecology, Medical University of Vienna, Vienna, Austria; 2 Department of Emergency Medicine, Medical University of Vienna, Vienna, Austria; 3 Division of Nephrology and Dialysis, Department of Medicine III, Medical University of Vienna, Vienna, Austria

## Abstract

The significance of antibodies directed against paternal epitopes in the context of obstetric disorders is discussed controversially. In this study anti-HLA and anti-MIC-A antibodies were analysed in sera of women with uneventful pregnancy (n = 101), preeclampsia (PE, n = 55) and gestational diabetes (GDM, n = 36) using antigen specific microbeads. While two thirds of the women with uneventful pregnancy or GDM were HLA and MIC-A antibody positive in gestational week 11 to 13 with a modest increase towards the end of pregnancy, women with PE showed an inverse kinetic: 90% were HLA antibody positive in gestational week 11 to 13 and only 10% showed HLA reactivities at the end of the pregnancy. HLA antibody binding strength was more pronounced in gestational week 14 to 17 in patients with PE compared to women with uneventful pregnancy (maximum median fluorescence intensity of the highest ranked positive bead 7403, IQR 2193–7938 vs. 1093, IQR 395–5689; p = 0.04) and was able to predict PE with an AUC of 0.80 (95% CI 0.67–0.93; p = 0.002). Our data suggest a pathophysiological involvement of HLA antibodies in PE. HLA antibody quantification in early pregnancy may provide a useful tool to increase diagnostic awareness in women prone to develop PE.

## Introduction

The frequent occurrence of circulating antibodies directed against paternal alloantigens during pregnancy has been discovered already more than 50 years ago^1,2^. However, the pathogenic relevance and the longitudinal evolution of such alloreactivity in relation to the course of normal or complicated pregnancy and delivery are still controversially discussed^3^. Our current knowledge is mainly based on the results of retrospective cross-sectional studies, many of them using cell-based antibody detection techniques. Depending on the sensitivity of employed assay systems, human leukocyte antigen (HLA) antibodies have been described for 6 to 50% of uneventful pregnancies^4–9^. While some authors have reported their first appearance in the 3^rd^ trimenon^7^, others have shown a peak already in the 1^st^ and 2^nd^ trimenon^10^, or even constant levels during the whole pregnancy^5^. A common finding has been a marked decline of leukocyte antibodies post-partum^6,8^. One study suggested even complete antibody clearance soon after delivery^7^, whereas others have provided evidence that low-level alloreactivity can persist for decades^11,12^. Recent studies using highly sensitive and specific solid phase HLA-specific assays have described an association of spontaneous preterm birth and fetal death with the trias of HLA antibody positivity in peripheral blood, chorioamnionitis and complement split product deposition at the feto-maternal interface^13–16^. Complement deposition at the syncytiotrophoblast has also been found in women with preeclampsia (PE)^17^. Finally, a relationship between HLA antibody formation and gestational diabetes (GDM) has been described^18^. The pathomechanisms underlying these reported associations are not well understood. One may hypothesize a direct contribution of HLA antibodies to endothelial dysfunction, a key component of both PE^19^ and GDM^20^. In addition, vascular pregnancy diseases including intrauterine growth retardation, intrauterine fetal death and PE have been associated with the detection of soluble major histocompatibility complex class I related chain (MIC)^21^ and endothelial shedding of MIC has been proposed as a novel physiological mechanism of fetal allograft immune escape by silencing maternal natural killer (NK) cells^22^. Considering recent data obtained in organ transplant recipients^23^, one may speculate that antibodies towards MIC promote endothelial damage also in the setting of pregnancy.

The present study was designed to (i) determine and characterize the occurrence of anti-HLA and anti-MIC-A antibodies in a cohort of women during pregnancy and after delivery in prospectively and longitudinally collected sera using a sensitive and antibody specific bead-array based solid phase assay system (ii) analyse anti-HLA and anti-MIC-A alloreactivity in uneventful pregnancies as compared to pregnancies complicated by PE or GDM, (iii) test associations of anti-HLA and anti-MIC-A antibodies with fetal pregnancy outcomes.

## Results

### Patient characteristics

This study included 1047 serum samples of 101 healthy women who had uneventful pregnancies, 55 women who developed PE, and 36 women with GDM, obtained during pregnancy and after delivery. Within the PE group longitudinal samples were available in 11 cases, 27% were early onset type of PE and 9% had intrauterine growth restriction (IUGR). For 44 women only one serum was available at a symptomatic state of PE shortly before the end of pregnancy, 66% were early onset type of PE and 43% had IUGR. Baseline characteristics of included women, in relation to obstetric complications are listed in Table 1. Compared to women with uneventful pregnancy, women with PE were older, more often nullipara, had a shorter duration of pregnancy, more often underwent caesarean section and delivered new-borns with a lower birth weight. Similar differences were noted comparing women with uneventful pregnancy and subgroups of women with PE with few exceptions. There was no difference in age and the ratio of nullipara between women with PE and longitudinal sampling (n = 11) and women with uneventful pregnancy. However women with PE had a higher body mass index (BMI). Likewise age lost level of significance in the group of women with PE at ‘state of disease’ (n = 44). However women with PE were more likely to be primigravidae and the number of gravidities and parities was lower. Women with GDM were older, had a higher BMI, more prior parities and a shorter duration of pregnancy, and delivered by caesarean section more often than women with uneventful pregnancies.

**Table 1:** Clinical baseline characteristics for women with uneventful pregnancy, women with PE and women with GDM.

		Uneventful pregnancy (n = 101)	PE (n = 55)	P-value^1^	PE longitudinal (n = 11)	P-value^2^	PE state of disease (n = 44)	P-value^3^	GDM (n = 36)	P-value^4^
Age, years	median (IQR)	30 (26–34)	34 (27–37)	0.02	34 (31–35)	0.07	34 (26–37)	0.07	34 (29–37)	0.03
Caucasian	%	94	98	0.42	100	0.41	98	0.34	94	0.93
BMI, kg/m^2^	median (IQR)	24 (22–27)	26 (23–30)	0.09	27 (26–30)	0.02	25 (23–29)	0.34	27 (24–34)	0.002
Gravidity, n	median (IQR)	2 (1–3)	2 (1–3)	0.22	2 (2–4)	0.16	1 (1–3)	0.04	3 (1–4)	0.07
Primigravidae	%	28	46	0.25	9	0.18	55	0.002	25	0.75
Multigravidae	%	35	31	0.64	46	0.48	27	0.38	53	0.06
Parity, n	median (IQR)	1 (0–1)	0 (0–1)	0.21	1 (0–2)	0.24	0 (0–1)	0.05	1 (0–2)	0.03
Nullipara	%	41	58	0.04	28	0.39	66	0.005	28	0.17
Multipara	%	7	13	0.23	9	0.79	14	0.19	22	0.01
Prior abortion	%	27	22	0.57	27	0.97	21	0.42	19	0.5
Prior abruption	%	3	7	0.24	18	0.08	5	0.63	6	0.61
Smoking	%	21	9	0.07	9	0.35	9	0.09	17	0.59
Pregnancy duration, days	median (IQR)	278 (269–284)	239 (207–259)	<0.001	256 (228–268)	<0.001	234 (207–248)	<0.001	269 (264–278)	<0.001
Caesarean section	%	36	87	<0.001	100	<0.001	84	<0.001	78	<0.001
Preterm delivery^5^	%	0	51	<0.001	27	0.001	57	<0.001	0	<0.001
Birth weight, g	median (IQR)	3410 (3160–3740)	1608 (990–2370)	<0.001	2310 (1530–2640)	<0.001	1530 (983–2228)	<0.001	3285 (2951–3668)	0.2
Infant sex female	%	52	55	0.74	36	0.34	59	0.39	42	0.34

BMI, body mass index; GDM, gestational diabetes; IQR, inter quartile range; PE, preeclampsia.

^1^uneventful pregnancy vs. PE; ^2^uneventful pregnancy vs. PE longitudinal. ^3^uneventful pregnancy vs. PE ‘state of disease’. ^4^uneventful pregnancy vs. GDM. ^5^(<week 34 + 0).

### Kinetics of HLA antibodies in women with uneventful pregnancies

**Figure 1:**
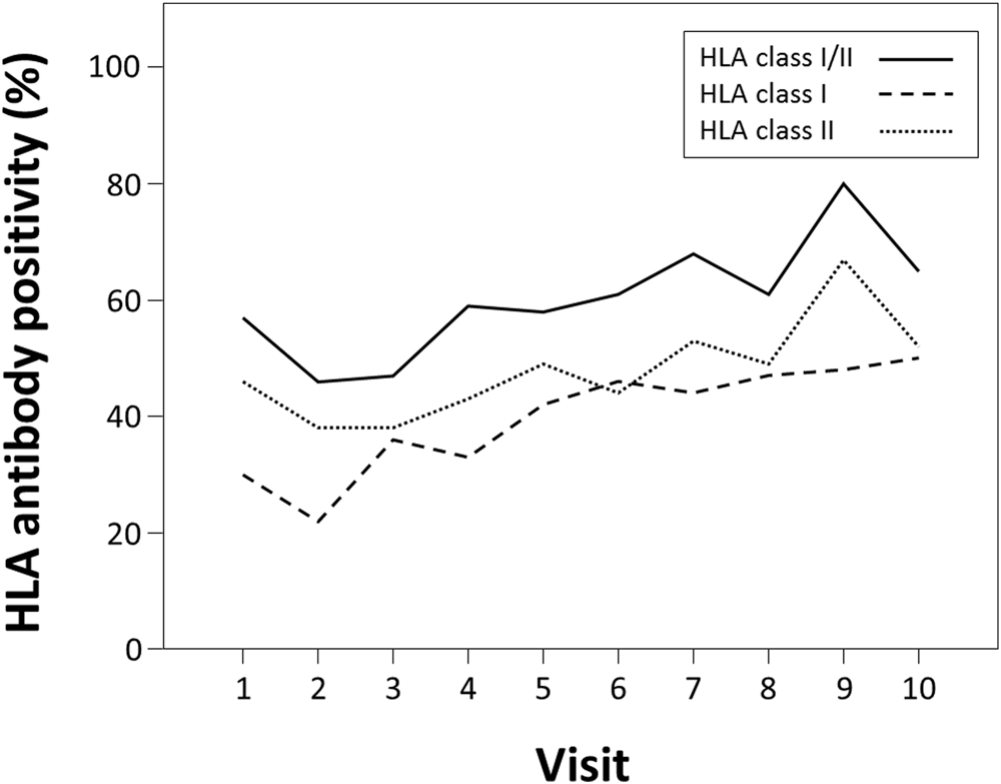
HLA antibody positivity in women without pregnancy complications, during the pregnancy (visit 1 to 7) and after delivery (visit 8 to 10; solid line, HLA class I and/or II antibody positive; dotted line, HLA class I antibody positive; dashed line, HLA class II antibody positive).

**Figure 2:**
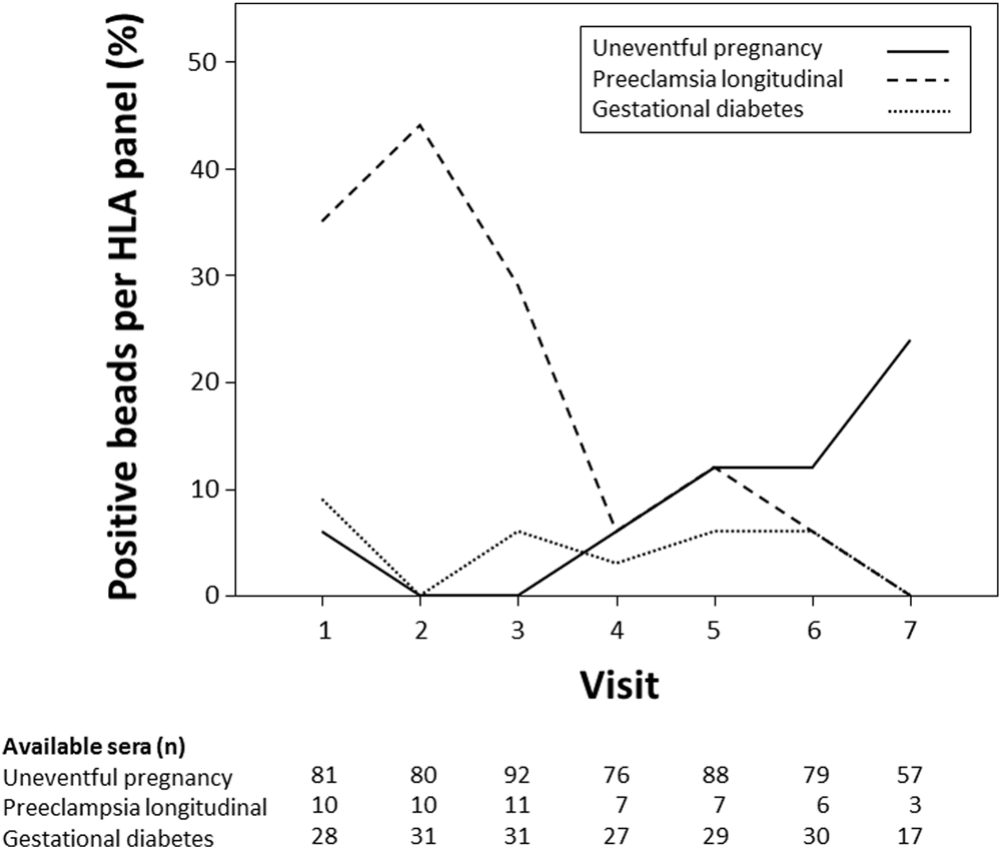
Median percentage of positive beads within the HLA bead panel per serum in the cohort of women without pregnancy complications (solid line), in women with PE (dashed line) and in women with GDM (dotted line) during the pregnancy (visit 1 to 7).

First, 101 women with uneventful pregnancies were tested for the presence of HLA alloreactivity using a Luminex-based solid phase screening assay, which covers HLA antibodies towards all major classical HLA antigens (HLA A, B, C, DR, DQ and DP). The majority of the women (n = 84, 83%) showed detectable HLA class I and/or class II reactivity during pregnancy, whereby 72% showed reactivity against HLA class I and 56% against HLA class II antigens. After delivery 70% (60% HLA class I, 50% HLA class II) still had detectable levels of HLA antibodies. At the first visit, already two thirds of the sera were tested antibody positive, with a modest increase over the course of pregnancy (Supplementary Table 1, Fig. 1). After delivery, an initial increase in reactivity was followed by a subsequent decrease in HLA antibody positivity. After six months (median 175 days after delivery, IQR 159–206), less than 60% of the women showed detectable reactivity. Kinetics were similar for HLA I and HLA II classes (Supplementary Table 1, Fig. 1). Analysing the broadness of HLA sensitization, we found that the median percentage of positive beads within the bead panel per serum was 6% (IQR 0–44%) during pregnancy. Low percentages were calculated for the first and second trimenon with a constant increase in the third trimenon and the highest values before delivery (Fig. 2).

### Association of HLA antibodies with clinical baseline data in women with uneventful pregnancies

Within the cohort of women with uneventful pregnancy, HLA antibody-positive and -negative women differed with respect to the number of gravidities and parities (Table 2). During pregnancy, 68% of primigravidae (n = 28) developed detectable HLA reactivity as compared to 82% of the women with a second (n = 38), 90% with a third gravidity (n = 19) and 100% with more than three gravidities (range 4 to 7, n = 16), respectively. As shown in Table 3, primigravidae were less often HLA antibody-positive than primi-/multipara. Moreover primigravidae had less positive beads per panel and, representing antibody binding strength, a lower median fluorescence intensity (MFI) of the highest ranked positive bead in the bead panel (MFImax) compared to primi-/multipara (Table 3). All 21 women who smoked had detectable HLA reactivity during the pregnancy compared to 78% of the women who did not smoke (Table 2). Women who smoked showed a higher percentage of positive beads per panel (18%, IQR 0–71% vs. 6%, IQR 0–41%; p = 0.008) and a trend towards a higher MFImax in HLA positive sera (2681, IQR 453–9847 vs. 729, IQR 314–3498; p = 0.08) over the whole pregnancy periode. As shown in Table 2, there was no difference in relation to the mode of delivery.

**Table 2:** Baseline characteristics of women without obstetric complications in relation to HLA antibody positivity.

		HLA antibody positive (n = 83)^1^	HLA antibody negative (n = 18)	P-value
Age, years	median (IQR)	30 (27–34)	28 (24–33)	0.08
BMI, kg/sqm	median (IQR)	24 (21–27)	26 (22–29)	0.21
Gravidity, n	median (IQR)	2 (2–3)	2 (1–2)	0.005
Parity, n	median (IQR)	1 (0–1)	0 (0–1)	0.03
Prior abortion	%	17	29	0.29
Caesarean section	%	39	40	>0.99
Smoking	%	25	0	0.016

BMI, body mass index; IQR, inter quartile range.

^1^HLA positivity during pregnancy (visit 1 to visit 7), for the variable 'caesarean section' HLA positivity after delivery (visit 8 to visit 10).

**Table 3:** HLA antibody characteristics of women with uneventful pregnancy in relation to gravidity and parity.

			Primigravidae (n = 28)	Primi-/multipara (n = 73)	P-value
Gestation week 11–13	HLA class I and/or II positive	%	30	67	0.003
	HLA class I positive	%	26	53	0.03
	HLA class II positive	%	17	43	0.03
	Percent positive beads per panel	median (IQR)	0 (0–6)	18 (0–43)	0.003
	MFImax	median (IQR)	257 (182–331)	2869 (2802–2936)	0.011
Whole pregnancy	HLA class I and/or II positive	%	68	88	0.02
	HLA class I positive	%	61	77	0.11
	HLA class II positive	%	43	62	0.09
	Percent positive beads per panel	median (IQR)	0 (0–18)	12 (0–64)	<0.001
	MFImax	median (IQR)	370 (277–1050)	3073 (509–7370)	<0.001

IQR, inter quartile range; MFImax, median fluorescence intensity of the highest ranked positive bead in the bead panel.

### HLA antibody kinetics in women with preeclampsia

**Figure 3:**
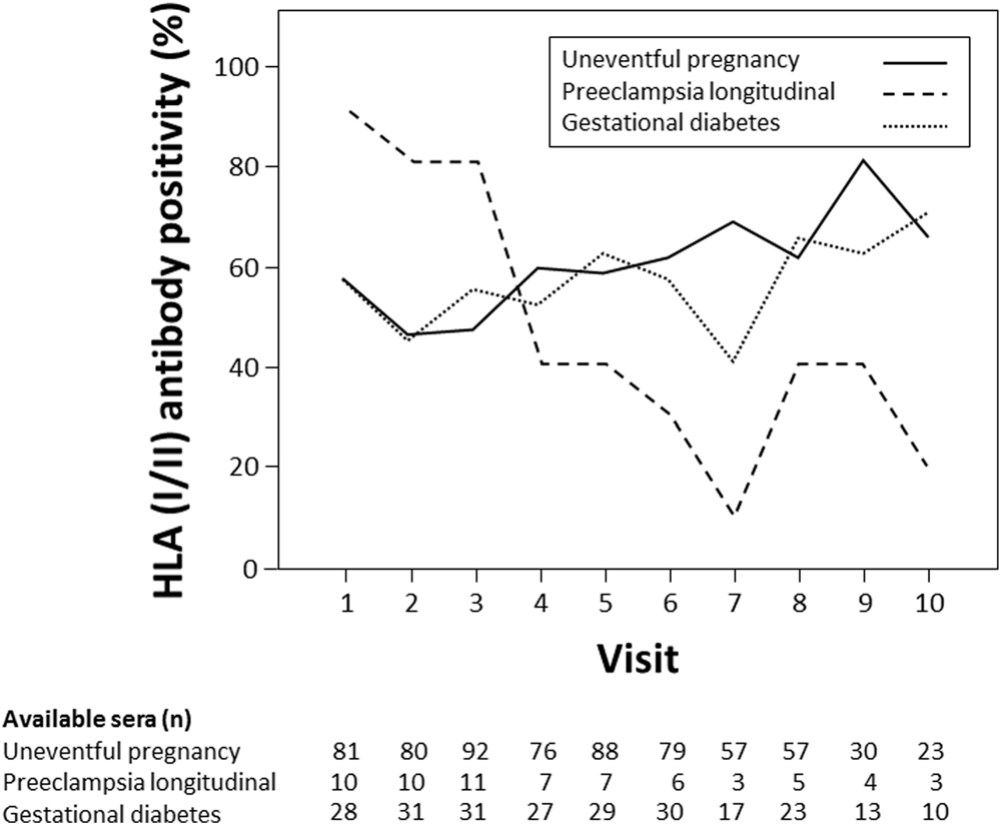
HLA I/II antibody positivity in cohorts of women without pregnancy complications (solid line), with PE (dashed line) and with GDM (dotted line) during the pregnancy (visit 1 to 7) and after delivery (visit 8 to 10).

Women with PE and prospectively and longitudinally collected sera (n = 11) or with a single serum drawn shortly before the end of pregnancy (n = 44) were tested for HLA antibodies. Analysing the 44 singleton sera of women with PE and the last available serum before delivery of the 11 women with PE and longitudinal follow-up, HLA positivity was detected in 64%, whereby 58% had reactivities directed towards HLA class I and 45% towards HLA class II. There was no difference in comparison to corresponding sera of healthy controls (HLA class I/II, p = 0.86; HLA class I, p = 0.42; HLA class II, p = 0.56). For analysis of HLA antibody kinetics, we restricted our observations to women with PE and longitudinal sampling (n = 11, median of 9 sera per individual). In contrast to women with uneventful pregnancy, almost all had detectable HLA reactivities (90%) at the first visit and HLA antibody positivity constantly decreased over time during pregnancy (10% after week 37; Supplementary Table 1, Fig. 3). Kinetics were similar for HLA I and HLA II classes (Supplementary Table 1). Median percentage of positive beads within the bead panel per serum during pregnancy was higher compared to healthy controls (21%, IQR 0–72% vs. 6%, IQR 0–44%; p = 0.03). In contrast to women with uneventful pregnancy women with PE had a high percentage of positive beads in the early pregnancy period with a constant decline towards delivery (Fig. 2). Similar to women with uneventful pregnancies, HLA antibody positivity was more frequent after delivery in women with PE.

### HLA antibody kinetics in women with gestational diabetes

A total of 69% of the women in the GDM group (n = 36, median of 7 samples per patient) had detectable HLA reactivities during pregnancy, which was not different compared to women with uneventful pregnancy (p = 0.075). Likewise women with GDM were similar to women without obstetric complication with respect to HLA class I (58%) and HLA class II (56%) reactivity during pregnancy (p = 0.12 and p = 0.92, respectively) and the percentage of positive beads within the bead panel per serum (6%, IQR 0–59%, p = 0.45). Kinetics of HLA alloreactivity was comparable to women with uneventful pregnancy (Supplementary Table 1, Fig. 3). There was also no difference in proportions of positive beads in the assay panel (Fig. 2).

### Quantitative and qualitative assessment of HLA antibodies in relation to obstetric disorders

**Figure 4:**
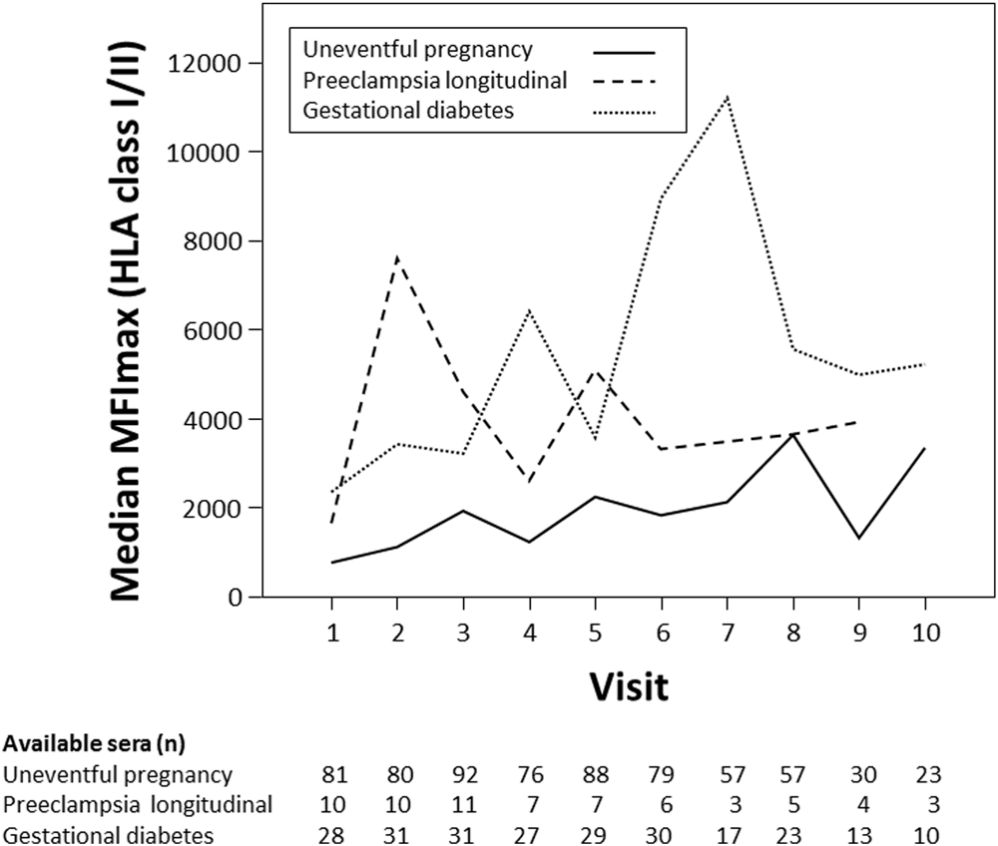
Median fluorescence intensity of the highest ranked positive bead in the HLA bead panel within the cohort of women without pregnancy complications (solid line), in women with PE (dashed line) and in women with GDM (dotted line) during the pregnancy (visit 1 to 7) and after delivery (visit 8 to 10).

As data derived from patients after solid organ transplantation have highlighted a predictive value of detecting HLA antibody binding strength for chronic alloreaction^24^, we analyzed the maximum of MFI in HLA antibody positive sera. Women with uneventful pregnancy had a median of 1675 MFImax (IQR 344–11812) during the whole pregnancy. MFImax was lowest at the beginning of the pregnancy, continually rose during the pregnancy and further increased shortly after delivery (Fig. 4). In contrast women with PE, for whom longitudinally collected sera were available (n = 11), showed the highest MFImax already in the early phase of the pregnancy with a peak at week 14 to 17 (Fig. 4), which was higher compared to women with uneventful pregnancy (7403, IQR 2193–7938 vs. 1093, IQR 395–5689; p = 0.04). Thereafter MFImax substantially decreased and remained low in the third trimenon and after delivery. Sera from week 14 to 17 were additionally tested by means of a single antigen bead test to determine antigen specificity on a single antigen level. Anti-HLA C reactivities were detected in all sera of the 80% of women who tested positive for HLA class I antibodies. In women with GDM MFImax showed a mean of 4228 during the whole pregnancy (IQR 1347–12122), which was significantly higher than in women with uneventful pregnancy (p < 0.001; Fig. 4). There was no significant difference in MFImax in the early phases of pregnancy compared to women with uneventful pregnancy. However, patients with GDM had significantly higher MFImax at the end of pregnancy (median 5669, IQR 1127–14314 vs. 1808, 359–8385; p = 0.037).

### HLA antibody binding strength for prediction of preeclampsia

**Figure 5:**
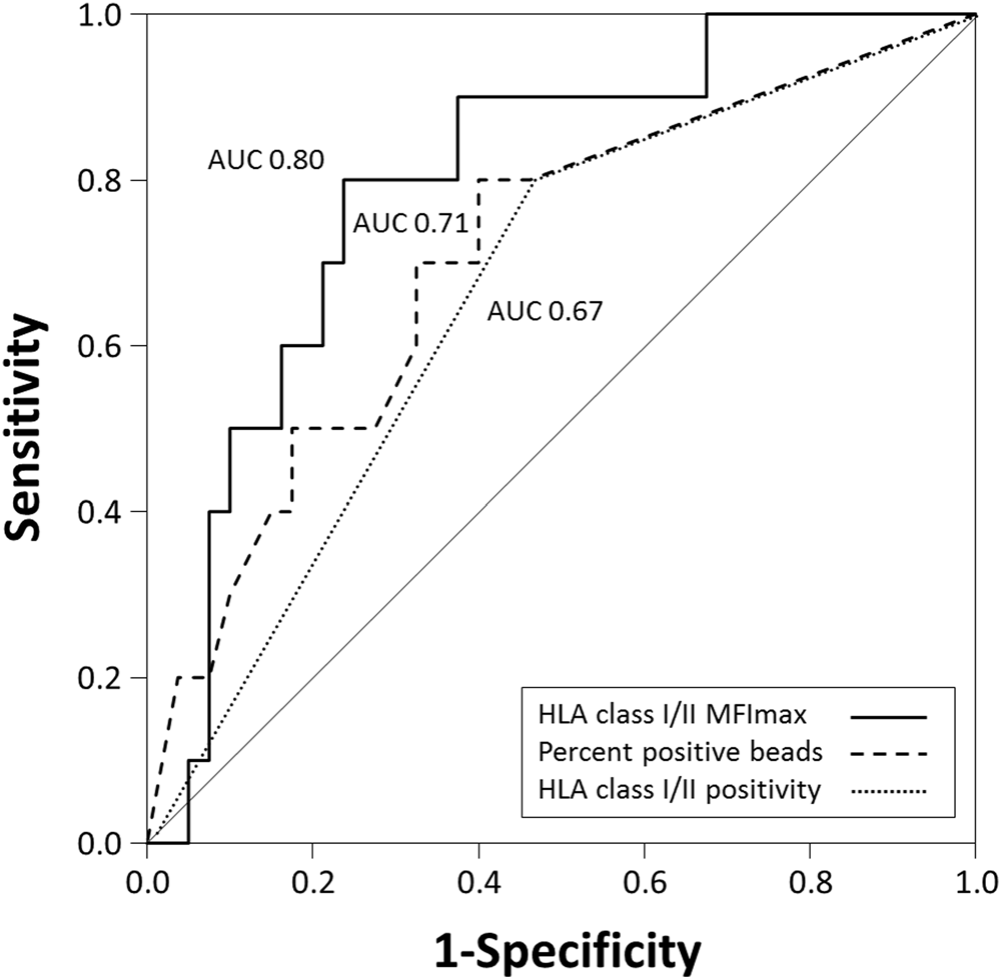
ROC and AUC for PE prediction at pregnancy week 14 to 17 using median of MFImax of HLA class I and/or II positive beads (solid line), percent of HLA class I and/or II positive beads per HLA panel (dashed line) and HLA class I and/or II antibody positivity (dotted line).

Quantitative assessment of HLA antibody binding strength (MFImax) reveals the most prominent differences between women with uneventful pregnancy and women with PE at an early stage of pregnancy. Analyzing the potency of HLA class I and/or II MFImax in PE prediction at gestational week 14 to 17, receiver operating characteristics (ROC), revealed an area under the curve (AUC) of 0.80 (95% CI 0.67–0.93, p = 0.002). AUC for MFImax performed better compared to AUC of HLA class I and/or II positivity (0.67, 95% CI 0.50–0–83, p = 0.08) and percent of HLA class I and/or II positive beads per panel (0.71, 95% CI 0.54–0.88, p = 0.03; Fig. 5). C-statistics for five representative values of MFImax are shown in Supplementary Table 2.

### HLA antibodies in relation to fetal characteristics

Next, we tested for associations between HLA reactivities and fetal characteristics in the group of women with uneventful pregnancy (n = 101). Offsprings of women who showed detectable HLA reactivity during pregnancy (n = 83), had a lower birth weight compared to women without antibodies (n = 18; 3400 gram, IQR 3080–3740 vs. 3623 gram, IQR 3320–3855, p = 0.039). Women with and without HLA antibodies had a similar pregnancy duration (277 days, IQR 269–282 vs. 279 days IQR 275–286, p = 0.118). The proportion of women having anti-HLA antibodies did not differ with regard to their offspring gender (83% with female vs. 82% with male offspring; p = 0.89).

### Major histocompatibility complex class I related chain A antibodies

**Figure 6:**
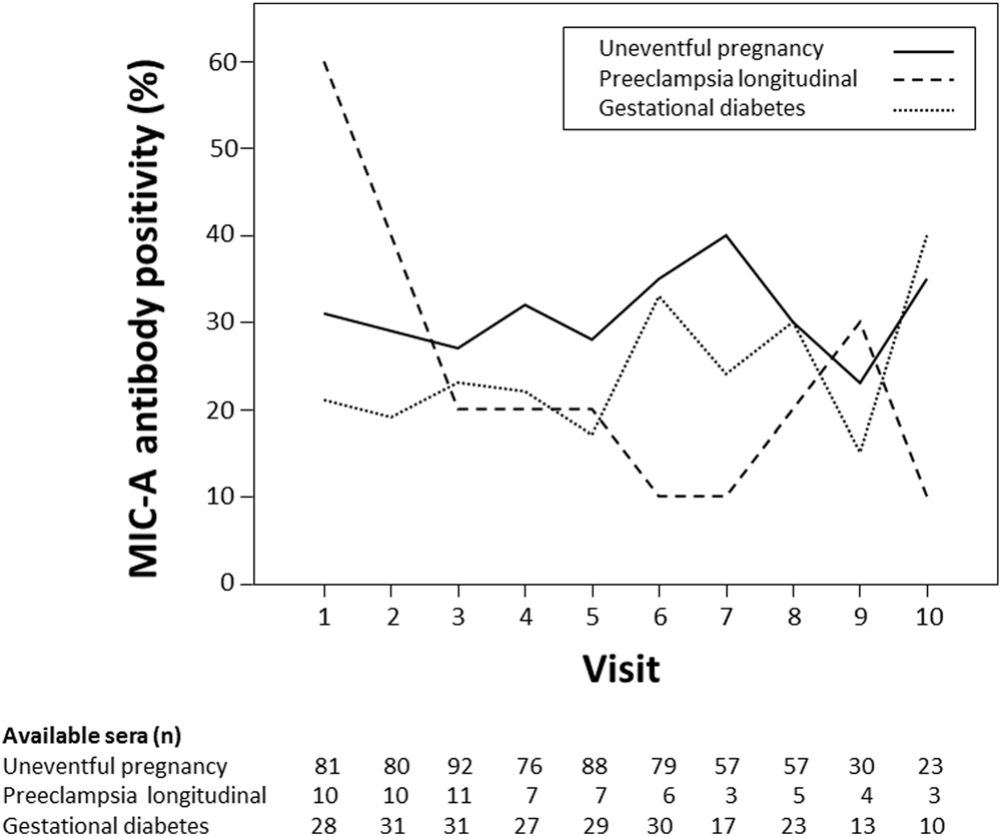
MIC-A antibody positivity in the cohort of women without pregnancy complications (solid line), in women with PE (dashed line) and in women with GDM (dotted line) during the pregnancy (visit 1 to 7) and after delivery (visit 8 to 10).

In women with uneventful pregnancies, antibodies directed towards MIC-A were detected in 53% during the pregnancy and 34% after delivery. MIC-A antibody kinetics showed an increase during pregnancy and a decrease after delivery (Fig. 6). In women with PE, MIC-A antibodies were detected in the majority of the women during the pregnancy and after delivery (73% and 67%, respectively). These percentages were not different compared to women with uneventful pregnancy (p = 0.22 and p = 0.23, respectively). In contrast to women with uneventful pregnancy MIC-A antibodies were highest at the beginning of the pregnancy with a constant drop towards the end of pregnancy (Fig. 6). In women with pregnancies complicated by GDM, levels and kinetics of MIC-A antibodies were similar to women with uneventful pregnancy (42% during the pregnancy, p = 0.22 and 28% after delivery, p = 0.5; Fig. 6). Within the group of women with uneventful pregnancy there was no difference in MIC-A antibody positivity with respect to pregnancy duration or offspring weight and sex (p = 0.5, p = 0.8 and p = 0.7, respectively).

## Discussion

This study was designed to determine the prevalence and kinetics of HLA and MIC-A antigen-specific antibodies in uneventful pregnancies and in pregnancies complicated by PE or GDM. A major finding was an inverse kinetics in pregnancies complicated by PE compared to women with uneventful pregnancy. Our data suggest that HLA antibody binding strength at gestational week 14 to 17 was predictive for the subsequent development of PE.

The physiologic relevance of HLA antibodies during pregnancy is unclear. Until now, most of the studies have used non-antigen specific assays and only cross sectional collected sera were analysed. We here provide the first report on HLA specific antibodies kinetics in women with uneventful pregnancies and women with PE analyzing longitudinally and prospectively collected sera. At the end of the first trimenon, half of the women with uneventful pregnancies were HLA antibody positive and subsequently showed a modest but constant increase in HLA reactivity during the pregnancy. In contrast, almost all patients who subsequently developed PE were HLA antibody positive at the end of the first trimenon and HLA reactivity gradually decreased towards delivery. Quantitative assessment of HLA reactivity in women who subsequently developed PE revealed a peak of HLA antibody intensity and broadness of sensitization at the beginning of the second trimenon with a constant decrease over time.

During early placentation, a subset of the extravillous trophoblast, the endovascular trophoblast (EVT), invades maternal vessels, partly displaces the maternal endothelium, and comes into direct contact with maternal blood in the lumen of spiral arteries. In PE, the interstitial invasion of the extravillous trophoblast into the uterine tissues is reduced and the intramural trophoblast and EVT exhibit an increased frequency of apoptosis in the walls of maternal spiral arteries^25,26^. EVT cells express fetal HLA G, E and F and classical HLA C, but no other classical HLA including HLA A, B, HLA DR, DQ and DP^27^. HLA sensitization is elicited by fetal nucleated cells, debris and microparticles that enter the maternal circulation during the pregnancy^3^. Previous studies have described anti HLA antibodies in half of the sera derived from women with uneventful pregnancies^4–9^, whereas our longitudinal analysis using a highly sensitive and HLA specific assay system detected HLA reactivities in the majority of women without obstetric complications. Such feto-maternal allo-recognition does not lead to rejection, but appears to facilitate the regular placental development by controlled inflammation^28^. This delicate balance is orchestrated by a multitude of finely regulated mechanisms of active tolerance towards the fetus^29^ including prevention of excessive antibody-mediated complement activation by upregulation of regulatory proteins^30^.

In the context of current literature two hypotheses emerge to explain the inverse kinetics of HLA antibodies in patients with PE described in our study. We hypothesize that the increased alloresponse in women with PE compared to women with uneventful pregnancy might contribute to an inadequate placental perfusion and endothelial dysfunction. High levels of HLA class I antibodies at the beginning of the pregnancy followed by a decrease during the pregnancy might correspond to an increased production of HLA C antibodies in the early phase of the pregnancy and binding at the EVT as pregnancy proceeds. In a subsequent step the complement system might be activated by the HLA antibodies on the EVT contributing to vascular alteration of maternal vessels. In this respect it is important to note that deposition of complement split product C4d, a surrogate of classical antibody mediated complement activation, is frequently found in placentas of patients with PE^30^. Interestingly, all women with PE in our cohort tested at week 14 to 17 had detectable alloantibodies directed to HLA-C. Given the missing expression of HLA II on EVT in healthy women and patients with PE^31^, HLA II antibodies might bind to circulating fetal cells or cross the placenta and bind to fetal endothelial cells^32^, contributing to morphological changes in the chorionic villi^33^. Alternatively, HLA antibody increase in the early phase of PE could just indicate increased feto-maternal allorecognition without pathophysiological contribution acting as an innocent bystander.

Although this is the first report on HLA antibody kinetics in women with PE, the role of such reactivity in pregnancy complications has already been investigated in previous studies. Kim and colleagues^17^ have analyzed HLA antibodies at the time of delivery or miscarriage and found no difference in HLA antibody positivity in women with PE compared to women with preterm delivery without PE. HLA antibodies were not associated with C4d staining of the syncytiotrophoblast. In this study, HLA antibody positivity was analyzed separately for PE subgroups with and without growth reduction, no control group of women without pregnancy disorders was included and another HLA assay was chosen to define HLA positivity. Such differences in study design might limit direct comparison with our report. Moreover, no longitudinal series of samples were tested. Given the marked changes of HLA antibody positivity over time described in our study, it is possible that analysis of HLA antibodies restricted to the time of miscarriage or delivery might miss associations of HLA reactivities and PE. In this respect it is important to note, that even in our analysis patients with PE and women without obstetric disorders showed a similar prevalence of HLA positivity at the end of pregnancy.

Within our cohort of women with uneventful pregnancy HLA antibodies were associated with a lower fetal weight. This observation adds to the emerging evidence pointing towards a causative role of HLA antibodies in spontaneous preterm birth and fetal death were a trias of HLA antibody positivity, chorioamnionitis and complement deposition at the feto-maternal interface has been described^13–16^.

In addition to HLA antibodies patients with PE showed an inverse kinetics of MIC-A antibodies compared to women with uneventful pregnancy. Of note, these differences did not reach level of significance. MIC is a stress-inducible surface protein that is expressed on a wide range of human cells including EVT^34^. MIC is modulating the function of NK cells, a major leukocyte subset involved in proper EVT invasion and spiral artery remodeling^35^. Recent studies suggest that elevated soluble MIC in maternal plasma of patients with PE impair NK-cell mediated vascular modeling^21^. In the setting of solid organ transplantation antibodies towards endothelial donor MIC have been associated with endothelial dysfunction^36^. In this context, the inverse kinetics of MIC-A antibodies described in our study might reflect pathophysiological relevant changes during PE.

Patients with GDM showed similar HLA antibody kinetics during pregnancy and after delivery compared to women with uneventful pregnancy. Whereas Steinborn and colleagues described a higher prevalence of HLA II antibodies in sera drawn at admission into the delivery room in patients with GDM using ELISA based HLA detection assay^18^, no such difference was noted in our cohort. The more sensitive micro bead based assay used in our study might account for such difference. Indeed, a higher prevalence of HLA II antibodies was noted in women with uneventful pregnancy as well as in patients with GDM compared to the work of Steinborn and colleagues in sera drawn shortly before the end of pregnancy. Quantitative antibody assessment in our cohorts revealed a higher intensity in the total of sera available over the whole pregnancy period in patients with GDM, but no significant difference was found in sera drawn at the early visits. Higher intensity of HLA antibodies were detected only in the last available sera before delivery or miscarriage. The underlying pathophysiology of GDM is unknown. It seems that GDM arises from a pathophysiology distinct from the abnormalities of type 1 and type 2 diabetes. Recent evidence suggests a role for metainflammation, or chronic, low-grade metabolically induced inflammation in GDM^37^. The increased HLA antibody intensity described in our study might reflect such inflammatory process.

Our study has several limitations. Due to the small sample size of women with PE and prospectively and longitudinal collected sera no multivariate analysis was performed. Our study design does not allow for differentiation of HLA specificity on an antigen level for the majority of the tested sera, because single antigen testing and fetal HLA phenotyping was not available. We could not perform histological analysis of the placenta in patients with PE, due to a lack of stored samples. Finally, our study cannot test for causality of HLA antibodies in the development of PE. Further studies will have to define HLA antibody specificity on a single antigen level in combination with maternal and fetal HLA typing and HLA expression on the surface of the different compartments within the feto-maternal interface to proof antibody specificity. In addition, HLA antibody kinetics will have to be analyzed in parallel with histological findings including complement deposition at the feto-maternal interface.

In conclusion, this study is the first to demonstrate an association of HLA antibody formation with PE. Most importantly our analysis provides a potential novel biomarker for an early prediction of PE. The value of HLA antibody binding strength quantification for PE prediction will have to be verified in a prospective setting.

## Materials and Methods

### Study subjects

For this single center case control study, prospectively collected serum samples and medical data were derived from the ‘Biobank for pregnancies at the Department of Obstetrics and Feto-maternal Medicine, Medical University of Vienna, Vienna General Hospital’ (‘Biobank’). The setup of the Biobank, which was established in 2007, has been previously described in detail^38^. Briefly, all patients undergoing prenatal care at the Department of Obstetrics and Feto-maternal Medicine were invited to participate in the longitudinal arm of the biobank. Infectious diseases and maternal age under 18 years were exclusion criteria. Peripheral blood and clinical data were collected at 11^th^–13^th^ (visit 1), 14^th^–17^th^ (visit 2), 18^th^–22^nd^ (visit 3), 23^rd-^27^th^ (visit 4), 28^th^–32^nd^ (visit 5), 33^rd^–36^th^ (visit 6), and after the 37^th^ (visit 7) week of gestation. In addition, participating women were invited to attend post-partal visits scheduled at 0–3 days (visit 8), 6–14 weeks (visit 9), and 3–9 months (visit 10) after delivery. The ‘state of disease arm’ of the Biobank additionally includes samples optained once only from patients at a symptomatic state of disease. Written informed consent was obtained from all participating women. Both the Biobank study and the present study were approved by the institutional review board of the Medical University of Vienna (EK number 619/2006 and 559/2015, respectively). All investigations were carried out in accordance with the relevant guidelines and regulations. After excluding participating women from selection if they presented with solid organ transplantation, malignancy, multiple pregnancies, chronic renal disease, rheumatic disease, pre-existing hypertension, proteinuria or diabetes, or with less than five out of ten possible completed Biobank visits, three groups were created for the present study: (i) women with the final outcome uneventful pregnancy (n = 101), (ii) women with the final outcome GDM (n = 36) and (iii) women with the final outcome PE (n = 55). For the women participating in the longitudinal arm of the Biobank, a median of seven sera from different time points was available. Of note, besides all women in the longitudinal arm of the Biobank with the final outcome of PE (n = 11), 44 women from the ‘state of disease arm’ of the Biobank were additionally included in the PE group. For these women, only one serum was available because they were included into the Biobank at a symptomatic state of PE shortly before the end of pregnancy (e.g. after being transferred from an external centre due to PE). The diagnosis of PE was based on high peripheral arterial blood pressure (two separate readings taken at least six hours apart of 140/90 mmHg or more) and ≥300 mg of protein (24-hour urine). Early-onset PE was defined as PE presenting before 34 + 0 weeks of gestation. IUGR was defined as an estimated fetal weight <5^th^ percentile combined with either a pulsatility index ≥95^th^ percentile in the arteria umbilicalis or new onset of oligohydramnion (amniotic fluid index ≤10^th^ percentile). Diagnosis of GDM was defined as insulin dependent glucose intolerance with onset or first recognition during pregnancy confirmed by abnormal oral glucose tolerance test. A multigravida was defined as a woman pregnant for at least the third time and a multipara as a woman who has born more than one child.

### HLA and MHC class I polypeptide-related sequence A assessment

Serum samples were stored at −80 °C and thawed immediately before laboratory analysis. HLA and MIC-A reactivities were determined using LABScreen Mixed Class I and II beads from lot number 20 (One Lambda, Canoga Park, CA, USA) in 96 well plates according to the manufacturers’ protocol. The reactivity of a test sample was calculated from the raw fluorescence values for each HLA coated bead. Anti-HLA serum reactivity was calculated by correcting the trimmed mean for non-specific binding to the negative control bead and background values to determine the normalized background ratio (NBG ratio). Five serum samples from non-transfused, non-transplanted male donors were tested to obtain an average background value. A positive reaction was defined by NBG ratios >2.2 as suggested by the manufacturer. The NBG ratio was calculated according to the manufacturers’ protocol. Each test run included a reference alloserum samples with defined HLA antibody specificity. In addition percentage of positive beads within the HLA beads panel and MFI of the highest ranked positive bead in the bead panel (MFImax) was recorded. Data acquisition was carried out on a Luminex 200 flow analyser (Luminex Corporation, Austin, TX). Specificity on a single antigen level was determined using the HLA class I single antigen bead assay (One Lambda; lot number 9) with a cut off of a trimmed mean MFI of 500. The assay was performed according to the manufacturers’ protocol.

### Statistical analysis

Summaries for continuous variables are presented as the median and IQR. The Mann-Whitney U test was used for comparing continuous data. Categorical variables are presented as absolute numbers and percentages, and group comparisons were made using the Chi-square or Fisher exact tests. A 2-sided P value less than 0.05 was generally considered statistically significant. Exact tests were used where applicable. ROC and their corresponding AUC values with a 95% confidence interval were calculated. We used MS EXCEL 2010 (Redmond, WA), IBM SPSS Statistics 20.0 (SPSS Inc., Chicago, IL) and STATA 14.1 (STAT coop., College Station, TX) for data management and analysis.

### Data availability

There are no restrictions on the availability of supporting data.

## Electronic supplementary material

Supplementary Dataset 1
