# Supplementary material for: Longitudinal assessment of HLA and MIC-A antibodies in uneventful pregnancies and pregnancies complicated by preeclampsia or gestational diabetes
Source: Sci Rep. 2017 Oct 19;7:13524. doi: 10.1038/s41598-017-13275-6 (PMC5648869; doi:10.1038/s41598-017-13275-6)
Supplement: Supplementary file 1 — Supplementary Dataset 1 [file 41598_2017_13275_MOESM1_ESM.doc]

**Longitudinal assessment of HLA and MIC-A antibodies in uneventful pregnancies and pregnancies complicated by preeclampsia or gestational diabetes**

Lorenz Kuessel^1^, Harald Herkner^2^, Markus Wahrmann^3^, Farsad Eskandary^3^, Konstantin Doberer^3^, Julia Binder^1^, Petra Pateisky^1^, Harald Zeisler^1^, Georg A. Böhmig^3^, Gregor Bond^3^*

^1^ Department for Obstetrics and Gynecology, Medical University of Vienna, Austria

^2^ Department of Emergency Medicine, Medical University of Vienna, Austria

^3^ Division of Nephrology and Dialysis, Department of Medicine III, Medical University of Vienna, Austria

*Correspondence and requests for materials should be addressed to Gregor Bond

Division of Nephrology and Dialysis, Department of Medicine III, Medical University of Vienna, Währinger Gürtel 18-20, A-1090, Vienna, Austria. Phone: +43-1-40400-43890; Fax: +43-1-40400-43860; E-mail: [gregor.bond@meduniwien.ac.at](mailto:gregor.bond@meduniwien.ac.at)

**Supplementary Table 1. Percentage of women with HLA antibody positive sera.**

| Visit number | 1 | 2 | 3 | 4 | 5 | 6 | 7 | 8 | 9 | 10 |
| --- | --- | --- | --- | --- | --- | --- | --- | --- | --- | --- |
| Gestation week  Post gestation | 11-13 | 14-17 | 18-22 | 23-27 | 28-32 | 33-36 | >37 | day 0-3 | week 6-14 | month 3-9 |
| Uneventful pregnancy (n=101) |  |  |  |  |  |  |  |  |  |  |
| HLA class I/II | 57 | 46 | 47 | 59 | 58 | 61 | 68 | 61 | 80 | 65 |
| HLA class I | 46 | 38 | 38 | 43 | 49 | 44 | 53 | 49 | 67 | 52 |
| HLA class II | 36 | 30 | 22 | 36 | 33 | 42 | 46 | 44 | 47 | 48 |
| PE (n=11) |  |  |  |  |  |  |  |  |  |  |
| HLA class I/II | 90 | 80 | 80 | 40 | 40 | 30 | 10 | 40 | 40 | 20 |
| HLA I class I | 70 | 80 | 70 | 30 | 40 | 30 | 10 | 40 | 40 | 20 |
| HLA II class II | 50 | 40 | 50 | 20 | 10 | 20 | 0 | 10 | 10 | 0 |
| GDM (n=36) |  |  |  |  |  |  |  |  |  |  |
| HLA class I/II | 57 | 45 | 55 | 52 | 62 | 57 | 41 | 65 | 62 | 70 |
| HLA I class I | 50 | 39 | 45 | 41 | 45 | 43 | 35 | 52 | 46 | 50 |
| HLA II class II | 43 | 39 | 39 | 37 | 48 | 47 | 29 | 61 | 54 | 60 |

GDM, gestational diabetes; PE, preeclampsia.

**Supplementary Table 2. C-statistic for five representative values**

**of HLA MFImax at gestation week 14 to 17 for PE detection.**

| MFImax^1^ | Sensitivity (%) | Specificity (%) | PPV (%) | NPV (%) |
| --- | --- | --- | --- | --- |
| 382 | 90 | 63 | 23 | 98 |
| 906 | 80 | 77 | 29 | 97 |
| 1972 | 70 | 79 | 29 | 96 |
| 2706 | 60 | 84 | 32 | 94 |
| 6805 | 50 | 90 | 38 | 94 |

NPV, negative predictive value; PPV, positive predictive value

^1^HLA class I and/or II MFImax at week 14 to 17
